# Structural and Functional Characterization of the ABA-Water Deficit Stress Domain from Wheat and Barley: An Intrinsically Disordered Domain behind the Versatile Functions of the Plant Abscissic Acid, Stress and Ripening Protein Family

**DOI:** 10.3390/ijms22052314

**Published:** 2021-02-26

**Authors:** Ines Yacoubi, Karama Hamdi, Patrick Fourquet, Christophe Bignon, Sonia Longhi

**Affiliations:** 1 Biotechnology and Plant Improvement Laboratory, Centre of Biotechnology of Sfax (CBS), University of Sfax, Street Sidi Mansour Km 6, Sfax 3018, Tunisia; hamdikarama06@gmail.com; 2 INSERM, Centre de Recherche en Cancérologie de Marseille (CRCM), Centre National de la Recherche Scientifique (CNRS), Marseille Protéomique, Institut Paoli-Calmettes, Aix-Marseille University, 27 Bvd Leï Roure, CS 30059, 13273 Marseille CEDEX 09, France; patrick.fourquet@inserm.fr; 3 Lab. Architecture et Fonction des Macromolécules Biologiques (AFMB), UMR 7257, Aix-Marseille University and Centre National de la Recherche Scientifique (CNRS), 163 Avenue de Luminy, Case 932, 13288 Marseille CEDEX 09, France; christophe.bignon@univ-amu.fr

**Keywords:** Pfam 02496, ABA-WDS domain, ASR (abscissic acid stress ripening protein), durum wheat, *Hordeum vulgare*, chaperone, intrinsic disorder, induced folding, circular dichroism

## Abstract

The ASR protein family has been discovered thirty years ago in many plant species and is involved in the tolerance of various abiotic stresses such as dehydration, salinity and heat. Despite its importance, nothing is known about the conserved ABA-Water Deficit Stress Domain (ABA-WDS) of the ASR gene family. In this study, we characterized two ABA-WDS domains, isolated from durum wheat (TtABA-WDS) and barley (HvABA-WDS). Bioinformatics analysis shows that they are both consistently predicted to be intrinsically disordered. Hydrodynamic and circular dichroism analysis indicate that both domains are largely disordered but belong to different structural classes, with HvABA-WDS and TtABA-WDS adopting a PreMolten Globule-like (PMG-like) and a Random Coil-like (RC-like) conformation, respectively. In the presence of the secondary structure stabilizer trifluoroethanol (TFE) or of increasing glycerol concentrations, which mimics dehydration, the two domains acquire an α-helical structure. Interestingly, both domains are able to prevent heat- and dehydration-induced inactivation of the enzyme lactate dehydrogenase (LDH). Furthermore, heterologous expression of TtABA-WDS and HvABA-WDS in the yeast *Saccharomyces cerevisiae* improves its tolerance to salt, heat and cold stresses. Taken together our results converge to show that the ABA-WDS domain is an intrinsically disordered functional domain whose conformational plasticity could be instrumental to support the versatile functions attributed to the ASR family, including its role in abiotic stress tolerance. Finally, and after validation in the plant system, this domain could be used to improve crop tolerance to abiotic stresses.

## 1. Introduction

Crop production must increase to meet population feed global demand that is estimated to increase continuously. It is well known that abiotic stresses such as extremes in temperature, drought, salinity, heavy metal concentrations, and radiation represent the most limiting factors for agricultural productivity worldwide [1]. To cope with such challenges, plants developed some adaptation strategies to escape the changing environmental conditions. Abiotic stress responses in plants are complex due to the interrelationship of mechanisms and variability of genes involved. Gene discovery, with the genomic resources developed recently for wheat, has enabled rapid assessment of gene function and directly accelerates efficient wheat breeding.

Back in 1993, the Abscisic acid, Stress and Ripening (ASR) gene was first identified in tomato from a cDNA library induced by abscisic acid (ABA) [2]. Over the past 30 years, many research studies have focused on the ASR gene family. Members of this family have been reported in many plant species ranging from gymnosperms to monocots and dicots [3]. This protein family is gaining importance and the number of identified ASR genes continues to increase. This gene family, which is conserved in the plant kingdom, is surprisingly not present in *Arabidopsis* and related species, suggesting that the *Brassicaceae* family possibly lacks their orthologous [4]. The ASR gene family is a key component of several regulatory networks [5]. Although the precise physiological function of the ASR gene family remains unknown, these genes are thought to have functional duality in plants. On one hand, functional characterization of *Salicornia brachiata* ASR1 (SbASR1) provided information that it may function as a transcription factor [6]. In the same context, tomato ASR1 can act as a transcription factor because of its ability to bind DNA in a sequence-specific and Zn-dependent manner [7,8]. On the other hand, ASRs have been shown to have chaperone-like activity for direct plant protection under stress conditions as in the case of ASR from wheat, plantain and tomato [3,7,9]. ASR proteins have been reported to be implicated in plant development, senescence, and fruit ripening and to play crucial roles in abiotic stress tolerance in most plants, as observed for *Oryza sativa* ASR1 (OsASR1) [10], *Zea mays* ASR1 (ZmASR1) [11], and *Triticum aestivum* (TaASR1) [12]. Most members of the ASR family have also been shown to be associated to abiotic stress tolerance and to be regulated by ABA [13]. The maize ASR1 (ZmASR1) acts both as a transcriptional regulator and as a chaperone-like protein [11]. The rice transcription factor ASR5, the ZmASR1 ortholog, plays multiple roles in response to drought stress by regulating ABA biosynthesis, promoting stomatal closure, as well as acting as a chaperone-like protein that possibly prevents drought stress-related proteins from inactivation [14]. As halophytes have a unique genetic with well-developed adaptation mechanisms to survive in a saline condition, an ASR gene (SbASR1) has been isolated and characterized from an extreme halophyte *Salicornia brachiata* [15]. The overexpression of SbASR1 in transgenic groundnut enhances the salinity and drought stress tolerance by functioning as a Late Embryogenesis Abundant (LEA) protein and a transcription factor [16]. Likewise, ASR from *Triticum aestivum* (TaASR1) over-expressed in tobacco plants increases tolerance to drought [12].

ASR proteins are highly hydrophilic, heat-stable plant tissue-specific DNA-binding proteins, for which structural data are scarce [17,18]. ASR proteins from many species were found to be disordered in their native state [7,13,17,19] and were hence classified within the family of intrinsically disordered proteins (IDPs), i.e., functional proteins that lack a stable 3D structure under physiological conditions [20]. Tomato ASR1 was reported to be an IDP in native conditions although environmental factors modulate its folding [19]. Likewise, we previously showed that durum wheat (*Triticum turgidum*, Tt) and barley (*Hordeum vulgare*, Hv) ASR1 proteins are intrinsically disordered and able to undergo a disorder-to-order transition under glycerol-induced dehydration and upon binding to zinc ions [17]. The conformational plasticity of ASR proteins is likely of physiological relevance [17].

Previous works illustrate the broad impact of intrinsic disorder in plant protein families in many areas of plant biology. Among these are the LEA proteins, a plant protein family with the largest number of known IDPs [21]. The importance of intrinsic disorder in LEA proteins is reflected in their striking functional versatility, a common characteristic of IDPs [22]. The ASR family was firstly proposed to be group 7 within the LEA superfamily [23,24]. Later on, and according to Hunault and Jaspard, the ASR family was classified as class 12 within the LEA proteins database (LEAPdb) [25]. In 2012 however, the same authors decided to exclude the ASR proteins from the LEA family [26]. The ASR family classification is still very ambiguous. In 2014, the group of Gonzalez supported the view that the ASR family should be classified as an important group of ABA-Water Deficit Stress Domain (ABA-WDS) proteins with distinct and unique functions and roles [3]. The conservation of the ABA-WDS domain (Pfam 02496 and IPR063494) in members of the ASR family (as illustrated in the multiple sequence alignment of ASR proteins we previously reported [9]) argues for a functional significance. Noteworthy, in some cases, as for instance in the ASR protein from rice (Os ASR1), the domain represents more than 65% of the full-length protein [27].

Domains represent one of the most useful levels to understand protein function, and domain family-based analysis has a profound impact on the functional characterization and annotation of recently released genome data. To the best of our knowledge, the conserved ABA-WDS domain of this protein family has never been experimentally investigated. Although the ASR gene family has been extensively explored in many plant species, the precise physiological function, mechanism of action and classification of ASR proteins remain largely unknown. In addition, experimental and functional data on the ABA-WDS domain in isolation are lacking, while they are of paramount importance to illuminate the function of the ASR family in plant stress tolerance breeding. In order to fill in this gap in knowledge, and with the aim of ascertaining whether the ABA-WDS domain is functional on its own, we herein report the identification, structural and functional characterization of two ABA-WDS domains, TtABA-WDS and HvABA-WDS, isolated respectively, from salt-stressed durum wheat and barley leaves. The rationale for focusing on durum wheat and barley resides in pursuing and extending our previous studies that reported the characterization of two ASR proteins from these two cereals [17] and that showed the role of the ASR family in salt and drought tolerance in durum wheat [9].

Our results reveal that these domains are consistently predicted to be intrinsically disordered and further confirmed their disordered nature experimentally. In addition, we investigated the conformational plasticity of these two domains under external environmental factors, such as temperature and glycerol-induced water deficit. Finally, we show that the two domains are able to prevent the inactivation of the enzyme lactate dehydrogenase (LDH) under heat and desiccation stress and we provide evidence that their heterologous expression in yeast improves its tolerance to salt and heat-cold stress.

## 2. Results

### 2.1. Cloning of TtABA-WDS and HvABA-WDS cDNA and Disorder Prediction of the Encoded Protein Domains

Using primers designed from a previously reported multiple sequence alignment of plant full-length ASR proteins and conceived to amplify ABA-WDS domains [9], two cDNAs were amplified, one from salt-stressed durum wheat (Tt) and one from barley (Hv) leaves. The amplified cDNAs were subsequently cloned and sequenced. Analysis of the two deduced amino acid sequences against InterPro and Conserved Domains Database (CDD) confirmed that the two sequences (hereafter referred to as TtABA-WDS and HvABA-WDS) correspond to ABA-WDS domains.

**Figure 1:**
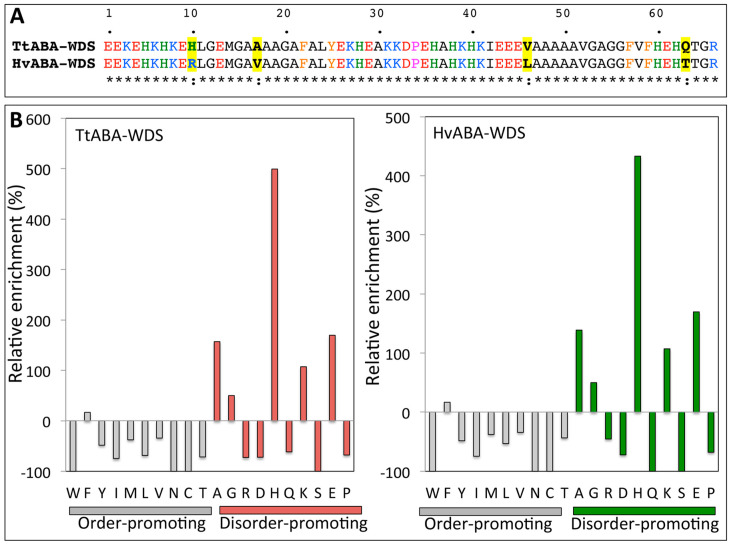
(**A**) Amino acid sequence alignment of TtABA-WDS and HvABA-WDS. Basic residues are in blue, acidic residues in red, prolines in pink, histidines in green and aromatic residues in orange. Amino acid substitutions are shown in bold on a yellow background. The alignment was generated with Clustal Omega and then edited manually. (**B**) Deviation in amino acid composition from the Swiss-PROT database of TtABA-WDS and HvABA-WDS. The relative enrichment in disorder promoting (pink and green bars) and depletion in order-promoting (grey bars) residues is shown. Residues have been ordered on the *x*-axis according to the TOP-IDP flexibility index as described in [28].

TtABA-WDS and HvABA-WDS cDNA encode polypeptides of 66 amino acids in length with a predicted molecular mass of ~7.2 kDa. The TtABA-WDS and HvABA-WDS sequences are very close to each other (94% of identity) and only differ in four amino acids (10H- > R; 17A- > V; 45V- > L; 63Q- > T, Figure 1A). The two domains’ acidic isoelectric points are 6.12 and 6.24, respectively (Table 1). Their predicted grand average of hydropathy index (GRAVY) values (as obtained from http://www.gravy-calculator.de/ (accessed on 25 January 2021)) are negative, underscoring their hydrophilic nature (Table 1).

The sequence composition of TtABA-WDS and HvABA-WDS domains were compared to that of proteins within the SWISS-PROT database (Figure 1B). Both domains have a biased sequence composition, being enriched in the most disorder-promoting amino acids (A, G, R, D, H, Q, K, S, E and P) and depleted in order-promoting residues (W, F, Y, I, M, L, V, C and T) [28]. Moreover, Ala (A), Glu (E) and His (H) are the most abundant amino acids in both domains. In line with these observations, TtABA-WDS and HvABA-WDS domains are predicted to be intrinsically disordered by the mean hydrophobicity/mean net charge ratio [29], as judged from their location in the left-hand side of the RH-plot (Supplementary Figure S1A,B).

**Figure 2:**
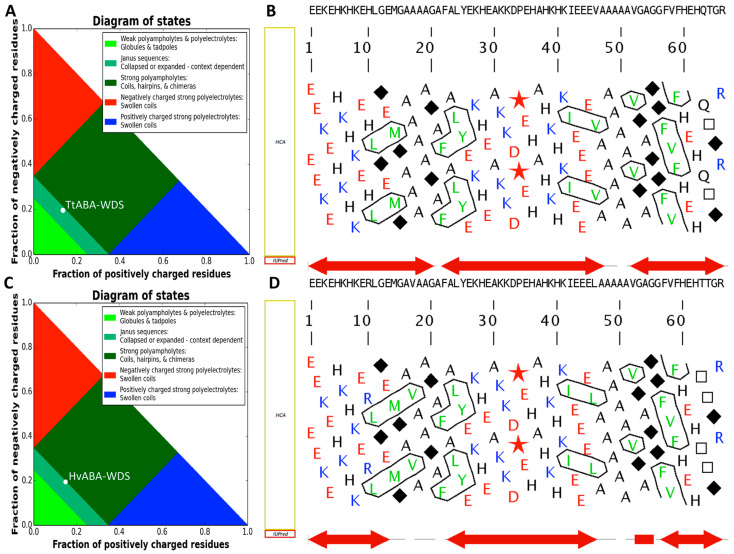
Phase diagram plots of TtABA-WDS (**A**) and HvABA-WDS (**C**) domains as provided by CIDER (http://pappulab.wustl.edu/CIDER/ (accessed on 25 January 2021)). MeDor [34] ouputs of TtABA-WDS (**B**) and HvABA-WDS (**D**) featuring the amino acid sequence above the HCA plot. The regions predicted as disordered by IUPRED [35] are represented by bi-directional red arrows.

The group of Pappu showed that sequence polarity is a determinant of IDP compaction, with polar IDPs having been found to favor collapsed ensembles in water despite the absence of hydrophobic groups [30]. To assess sequence polarity, the net charge per residue (NCPR, defined as |f_+_ − f_−_|) [30,31], the total fraction of charged residues (FCR, defined as f_+_ + f_−_), and linear distribution of opposite charges (κ value) [32] were calculated (Table 1). For both domains, the NCPR is negative, and the FCR values of TtABA-WDS and HvABA-WDS domains are close (0.333 and 0.348, respectively). According to the predictive diagram of states developed by Pappu and colleagues [33], both domains fall in phase diagram region (PDR) 2, with both being however very close to the boundary separating PDR2 and PDR3 (Figure 2A,C). PDR 2 corresponds to proteins that adopt conformations likely representing a continuum of possibilities between PDR 1, which accommodates weak polyampholytes or polyelectrolytes adopting a globular conformation, and PDR 3, which embeds strong polyampholytes adopting non-globular, swollen coil-like conformations. Both TtABA-WDS and HvABA-WDS domains have very low κ values indicating that opposite charges are well mixed and suggesting that these two domains adopt preferentially extended and swollen, coil-like conformation.

The amino acid sequences of HvABA-WDS and TtABA-WDS were analyzed also using the MeDor server for predicting protein disorder [34]. Hydrophobic Cluster Analysis (HCA) [36] (see Supplementary text) of the two domains shows that they are depleted in hydrophobic clusters (Figure 2B,D), a property indicative of protein disorder. The HCA plots of the two domains are very similar except for the size of the first hydrophobic cluster that encompasses two hydrophobic residues (LeuMet) in the case of TtABA-WDS, and three hydrophobic residues (LeuMetVal) in the case of HvABA-WDS (Figure 2B,D). This difference arises from the 17A- > V substitution. Additionally, the number of disordered residues predicted by IUPRED [35] is larger in TtABA-WDS than in HvABA-WDS (Figure 2B,D).

Analysis of the amino acid sequence of the two domains with the Phyre2 protein fold recognition server [37] returned a very low-confidence (~16%) model encompassing residues A_30_-V_52_, in which residues H_40_-A_51_ adopt an α-helical conformation (data not shown). Failure of the server to predict a model over most of the sequence, together with the very low confidence of the predicted α-helix, is consistent with the expectedly predominantly disordered nature of the domains.

### 2.2. Expression and Purification of HvABA-WDS and TtABA-WDS Domains

TtABA-WDS and HvABA-WDS cDNAs were cloned into the pGEX4-T1 expression vector to generate N-terminally glutathione S transferase (GST) tagged proteins. The two recombinant plasmids were then used to transform the *E. coli* strain BL21, where they were expressed as a major protein product (data not shown).

**Figure 3:**
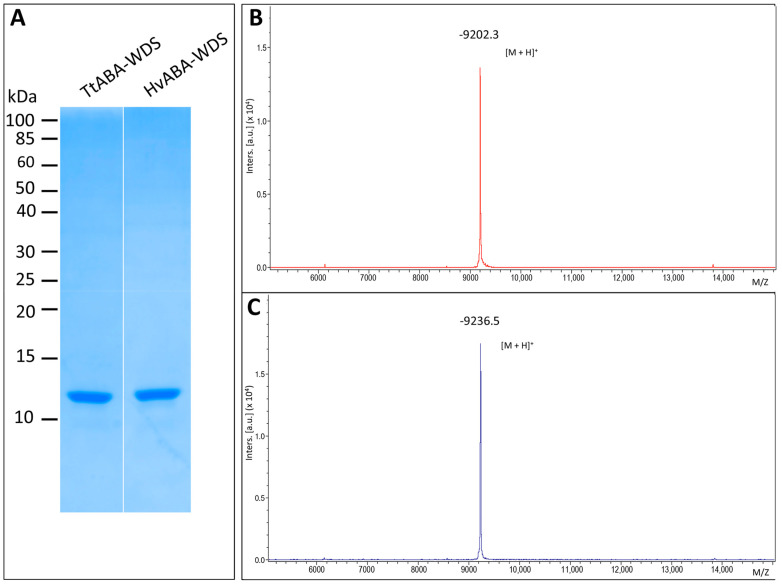
Purification of TtABA-WDS and HvABA-WDS domains from *E. coli*. (**A**) Coomassie blue staining of an 18% SDS-PAGE analysis showing the purified products as obtained after SEC. (**B**,**C**) MALDI-TOF-TOF mass analysis of intact TtABA-WDS (**B**) and HvABA-WDS (**C**) proteins. (**B**) Intact TtABA-WDS generated an average mass of 9201.30 Da for a theoretical mass of 9200.12 Da. (**C**) Intact HvABA-WDS generated an average mass of 9235.50 Da for a theoretical mass of 9234.22 Da. Masses were obtained with respectively 128 ppm (**B**) and 140 ppm (**C**) errors compatibles with the resolution of the spectrometer.

They were purified from the soluble fraction by GST affinity chromatography, followed by thrombin cleavage to remove the GST tag and size-exclusion chromatography (SEC). The final purified products (Figure 3A) exhibit an abnormally slow migration in SDS-PAGE: the apparent molecular mass (MM) of the recombinant domains, is estimated to be about 12 kDa, i.e., 1.3 times larger than that predicted by the amino acid sequence (~9.2 kDa, including the vector-encoded residues). Note that for both proteins, MALDI-TOF mass spectrometry (MS) analysis revealed the presence of a unique species with a molecular mass very close to the value calculated from the amino acid sequence (Figure 3B,C). In addition, MS analysis of peptides resulting from GluC digestion confirmed the identity of the purified proteins (Supplementary Figure S2). From MS analysis the purity of the proteins was estimated to be >95%.

IDPs often display an aberrant electrophoretic migration that is mainly due to their typical compositional bias, e.g., high net charge and low hydrophobicity that causes them to bind less SDS than globular proteins [38,39]. The anomalous electrophoretic behavior of TtABA-WDS and HvABA-WDS constitutes a hint of their likely disordered nature.

### 2.3. Hydrodynamiques Properties of HvABA-WDS and TtABA-WDS Domains from Size Exclusion Chromatography (SEC)

The Stokes radii (R_S_ of TtABA-WDS and HvABA-WDS domains were inferred using SEC. The elution profile of proteins in fact reflects their hydrodynamic properties. SEC was carried out using PBS pH 7.3 as elution buffer. TtABA-WDS and HvABA-WDS domains were eluted from the gel filtration column as sharp peaks (data not shown). From their elution volume (75.5 mL for HvABA-WDS and 72.1 mL for TtABA-WDS), their R_S_ were estimated to be 20.3 Å for HvABA-WDS and 21.5 Å for TtABA-WDS (Table 2).

By comparing the measured R_S_ (R_s_^obs^) for HvABA-WDS and TtABA-WDS with the theoretical Stokes radii expected for various conformational states (R_s_^NF^: natively folded protein; RsU: fully unfolded form; R_S_^IDP^: expected for an IDP), the two domains were found to have R_S_ values ~1.3 times larger than those of the corresponding R_s_^NF^ values, and close to the value expected for an IDP (Table 2). These results provide experimental evidence for the prevalently disordered nature of TtABA-WDS and HvABA-WDS. Their compaction index indicates that TtABA-WDS is more extended than HvABA-WDS (Table 2).

### 2.4. Differential Scanning Fluorimetry of HvABA-WDS and TtABA-WDS Domains

**Figure 4:**
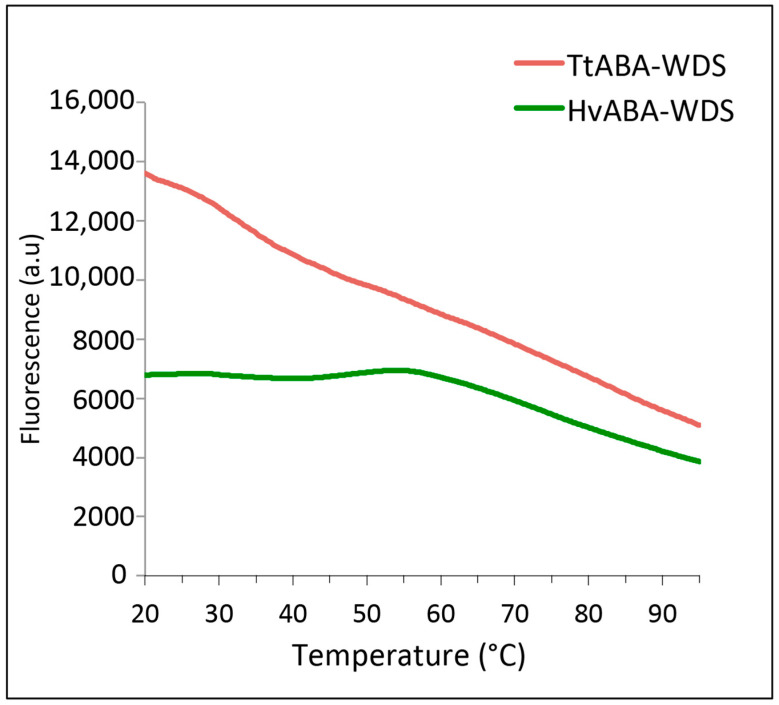
Differential scanning fluorimetry (DSF) of TtABA-WDS and HvABA-WDS domains in the presence of Sypro Orange in the 20–95 °C temperature range.

TtABA-WDS and HvABA-WDS were further analyzed by differential scanning fluorimetry (DSF), a technique used to determine the conformational stability of proteins that relies on the use of a fluorescent dye whose fluorescence is enhanced when protein hydrophobic cavities become accessible as a result of temperature-induced protein unfolding. IDPs, which do not possess hydrophobic cavities being devoid of a stable 3D structure, are characterized by a fluorescence profile that is rather flat and temperature-independent [17]. The experimentally observed profile obtained for HvABA-WDS is flat, consistent with the lack of a stable 3D structure (Figure 4). Albeit the profile of TtABA-WDS is not flat and rather features a progressive decrease in the fluorescence with increasing temperature, it still lacks the typical transition peak observed for a folded protein undergoing a cooperative transition (Figure 4). As such, the observed profile is indicative of the lack of 3D structure like in the case of HvABA-WDS. Therefore these results strengthen the conclusion that TtABA-WDS and HvABA-WDS are intrinsically disordered domains (IDDs), in line with in silico analyses and with the above-described experimental lines of evidence.

### 2.5. Circular Dichroism (CD) Studies of HvABA-WDS and TtABA-WDS

**Figure 5:**
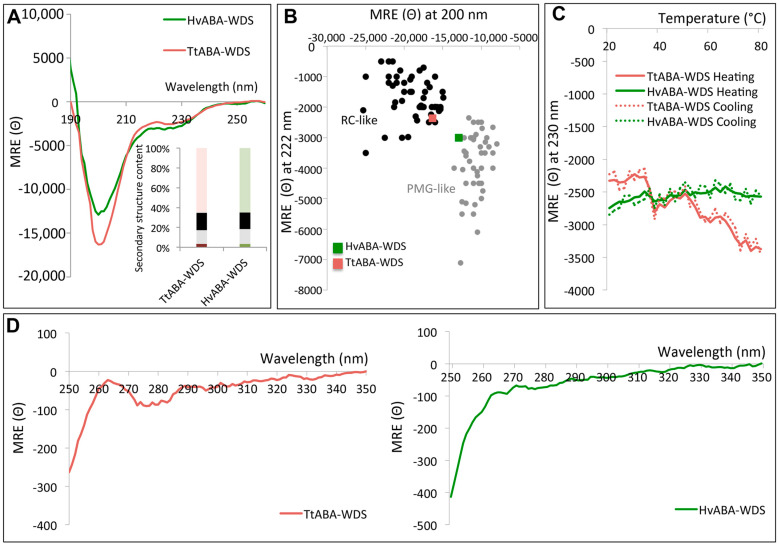
Analysis of TtABA-WDS and HvABA-WDS domains by circular dichroism. (**A**) Far-UV CD spectra of TtABA-WDS and HvABA-WDS at 0.12 mg/mL in 10 mM sodium phosphate pH 7 at 20 °C. Data are representative of one out of three independent measurements. The inset shows the secondary structure content of the two domains, as derived using CDSSTR. Light green or light red: unordered; black: turns; Green or red: helix; grey: strands. (**B**) Plot of the molar residue ellipticity (MRE) at 222 nm and at 200 nm of a set of well-characterized unfolded, random coil-like (RC-like) or PreMolten Globule-like (PMG-like) proteins (from [40]). The position in the plot of TtABA-WDS and HvABA-WDS is highlighted. (**C**) Molar residue ellipticity (MRE) at 230 nm of TtABA-WDS and HvABA-WDS as a function of the temperature. (**D**) Near-UV CD spectra of TtABA-WDS and HvABA-WDS at 1.2 mg/mL in 10 mM sodium phosphate pH 7 at 20 °C. Data are representative of one out of two independent acquisitions. MRE (Θ) is expressed in deg cm^2^ dmol^−1^.

To assess the secondary structure content of TtABA-WDS and HvABA-WDS, circular dichroism (CD) measurements were performed in the far-ultraviolet (UV) region. Under native conditions and neutral pH, both spectra display a large negative peak centered at 200 nm, low amplitude in the 220–230 nm region, and low ellipticity at 190 nm (Figure 5A). Both domains present spectra typical of disordered proteins lacking any stable organized secondary structure. Spectral deconvolution revealed a high content (about 50%) of unordered structure in both domains (Figure 5A, inset).

Previous studies unveiled that far-UV CD spectroscopy enables discriminating between IDPs adopting a random coil-like (RC) state and a premolten globule-like (PMG) state, based on the ratio of the ellipticity values at 200 and 222 nm [40]. PMGs possess some transiently populated secondary structure and are thus more structured than RCs. According to their ellipticity values at 200 and 222 nm, TtABA-WDS and HvABA-WDS domains can be classified in the RC-like and PMG-like region, respectively (Figure 5B). Thus, HvABA-WDS is more compact than TtABA-WDS, in agreement with SEC data that highlighted a larger compaction index (CI) for HvABA-WDS compared to TtABA-WDS (Table 2).

We next assessed whether the two domains undergo temperature-induced folding. To this end, we monitored the ellipticity at 230 nm as a function of temperature (Figure 5C). While TtABA-WDS undergoes temperature-induced folding, the profile of HvABA-WDS is roughly linear and devoid of a clear temperature-dependence (Figure 5C). To rule out any possible temperature-induced aggregation of the proteins, we monitored the HT voltage associated with the transmission of light through the protein in the CD spectropolarimeter during the heating of the proteins. The HT voltage is in fact extremely sensitive to changes in the level of scattering of transmitted light. Aggregation of protein at high temperature will lead to an increase in the HT voltage. We observed no significant changes in the HT voltage associated with heating indicating a lack of aggregation during heating (data not shown). In addition, we also monitored the absorbance at 340 nm (indicative of the presence of aggregates [41]) before and after heating the samples at 80 °C and observed no differences. We also monitored the ellipticity at 230 nm upon cooling the sample from 80 °C to 20 °C. The obtained profiles are almost indistinguishable from the heating profiles (Figure 5C) thereby attesting to the full reversibility of the process and hence definitively excluding possible irreversible aggregation-related processes.

To provide more evidence on the conformational state of TtABA-WDS and HvABA-WDS domains, we analyzed the environment of the aromatic amino acid side chains, which provides information about the tertiary structure, by measuring the CD spectra in the near-UV region (250–350 nm) at 20 °C [42]. Although the near-UV CD spectra of TtABA-WDS and HvABA-WDS are not superimposable, they are both characterized by a weak intensity and by the presence of a broad negative band (Figure 5D), once again consistent with the lack of a stable tertiary structure and of a hydrophobic core containing oriented aromatic residues as typically found in globular proteins [43]. The differences observed between the two near-UV CD spectra likely reflect differences between the two domains that are also pinpointed by far-UV CD studies.

### 2.6. TtABA-WDS and HvABA-WDS Domains Have an Intrinsic Propensity to Fold into an α-Helical Conformation

**Figure 6:**
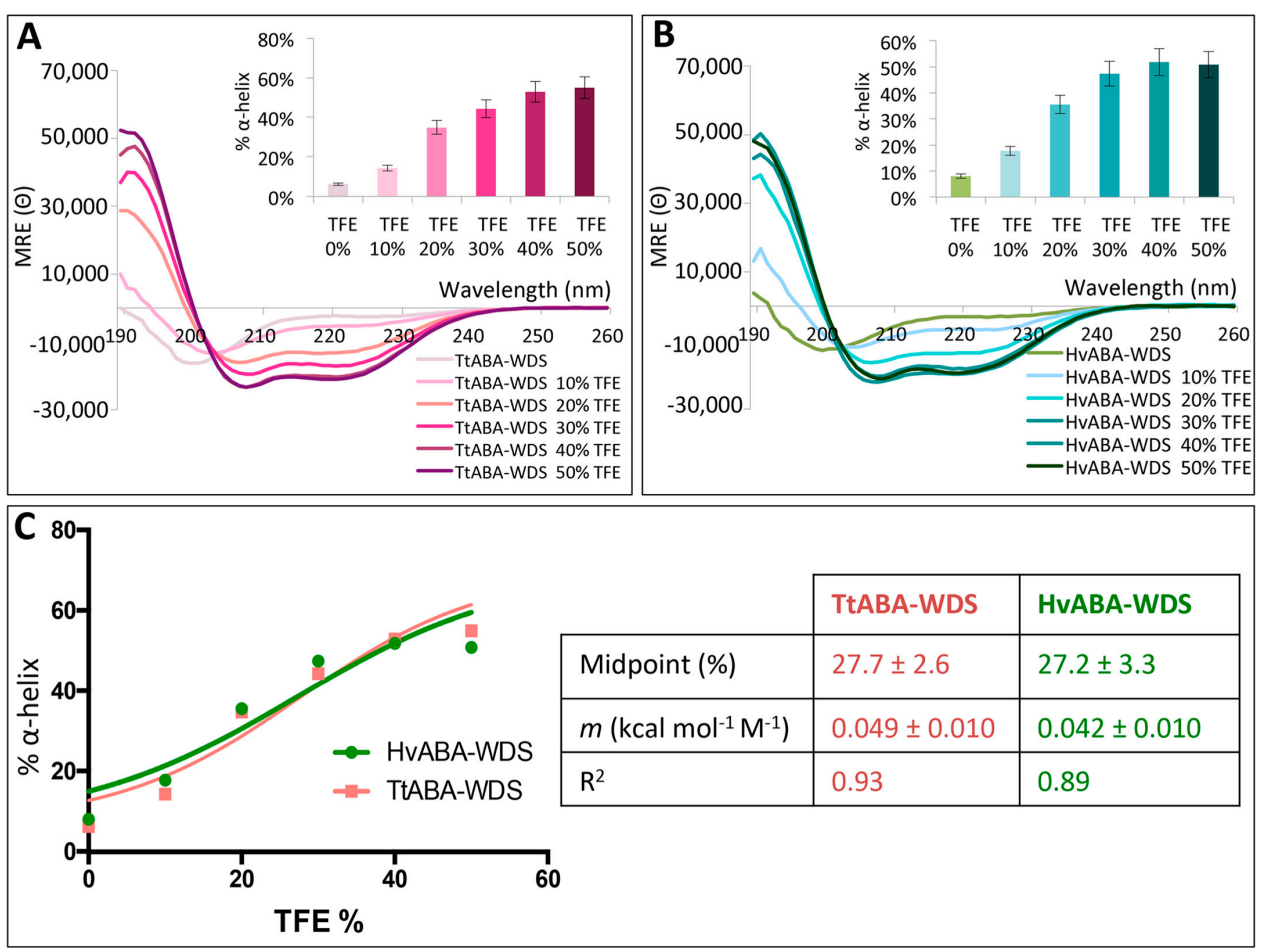
Far-UV CD spectra of HvABA-WDS (**A**) and TtABA-WDS (**B**) in the presence of increasing concentrations of TFE at 20 °C. Proteins were at 0.12 mg/mL in 10 mM sodium phosphate pH 7. Data are representative of one out of two independent acquisitions. The insets show the α-helical content as derived as described in Materials and Methods (see Equation (6)). MRE: molar residue ellipticity (Θ) in deg cm^2^ dmol^−1^. (**C**) Transition diagrams of HvABA-WDS and TtABA-WDS. The α-helical content of the two domains, as derived from the CD spectra, was plotted as a function of the TFE concentration and data were fitted to sigmoidal curve, corresponding to a two-state transition. The inset shows the midpoint of transition and *m* values, as derived from the fitting, along with the quality of the fitting.

Some of the most striking functional advantages of IDPs come from their ability to contextually change their conformation due to binding or environmental changes [44]. In order to assess the folding potential of TtABA-WDS and HvABA-WDS domains, their far-UV CD spectra were recorded in the presence of TFE. This organic solvent is a secondary structure stabilizer that is used to mimic the hydrophobic environment experienced by proteins during interactions [45,46] It is thus widely used as a probe of hidden structural propensities to unveil protein regions having a propensity to undergo induced folding [39,47]. For both domains, the addition of TFE induces a gain of α-helicity indicated by the characteristic maximum at 190 nm and double minima at 208 and 222 nm (Figure 6A,B). The estimated α-helical content gradually increases upon increasing the TFE concentration from 0 to 50% (Figure 6A,B and insets). For both proteins, the most important transitions take place at 30% of TFE. We noted also that for the two domains, the spectra display an isodichroic point at 202 nm indicative of a two-state transition (Figure 6A,B). We therefore plotted the percentage of α-helix as a function of the TFE concentration and fitted the data to a sigmoidal curve, corresponding to a two-state transition (Figure 6C). The fitting yielded a midpoint of transition at a TFE concentration of ~27% for both domains. From the fitting procedure, the *m* value could also be obtained. The latter is a measure of the osmolyte efficacy in folding or unfolding a protein. It also reflects the cooperativity of folding/unfolding in the presence of the osmolyte, and arises from the free energy contributions of protein groups that either become exposed upon unfolding or buried upon folding the protein. The *m* value is typically positive for protecting osmolytes, such as TFE, that drive the equilibrium towards the folded state. In agreement, *m* values of 0.049 kcal mol^−1^ M^−1^ and of 0.042 kcal mol^−1^ M^−1^ were obtained for TtABA-WDS and HvABA-WDS, respectively. The two domains therefore feature midpoints and *m* values very close to each other, indicating that they follow a very similar folding pathway.

### 2.7. TtABA-WDS and HvABA-WDS Domains Gain α-Helicity under Conditions Mimicking Dehydration

**Figure 7:**
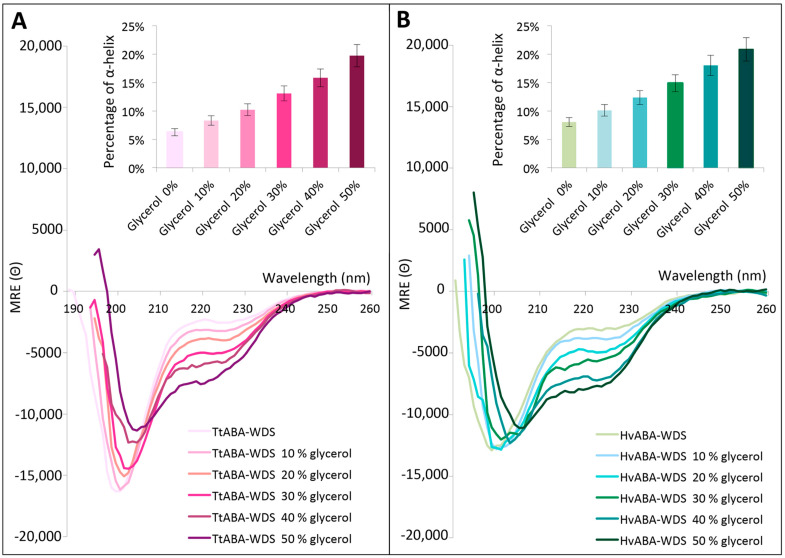
Far-UV CD spectra of HvABA-WDS (**A**) and TtABA-WDS (**B**) in the presence of increasing concentrations of glycerol at 20 °C. Domains were at 0.12 mg/mL in 10 mM sodium phosphate pH 7. Data are shown to the point up to which the dyna voltage was in the permissible range. Data are representative of one out of two independent acquisitions. The insets show the α-helical content as derived as described in Materials and Methods (see Equation (6)). MRE: molar residue ellipticity (Θ) in deg cm^2^ dmol^−1^.

Glycerol is known as a simplistic model for mimicking cellular dehydration that induces changes in the water surface tension around proteins. Here, we sought at assessing whether the two cereal domains could gain structure under increasing glycerol concentrations. The CD spectra of TtABA-WDS and HvABA-WDS domains show that the two domains fold in the presence of glycerol (Figure 7). Increasing the glycerol concentration triggers an increase in the α-helical content of both domains. Upon increasing the glycerol concentration from 0 to 50% the α-helical content augments from about 8% to 21% for HvABA-WDS and from about 6% to 20% for TtABA-WDS (Figure 7). The CD spectra of TtABA-WDS display an isodichroic point at around 208 nm. In the case of HvABA-WDS, the spectra, with the notable exception of the spectrum at 50% glycerol, exhibit an isodichroic point at 204 nm. The peculiar behavior of HvABA-WDS observed at 50% glycerol might reflect the fact that additional, different structural transitions take place under these conditions suggesting that the glycerol-induced structural transition experienced by this domain is more complex than a two-state transition.

The gain of structure observed in the presence of glycerol could arise from dehydration or from crowding effects (i.e., increased excluded volume effects). To discriminate between these two hypotheses, we also recorded the far-UV CD spectra of both domains in the presence of 30% of sucrose. The addition of sucrose does not induce a pronounced folding in the two domains and triggers only a slight increase in the α-helical content of TtABA-WDS (Supplementary Figure S3). Upon addition of sucrose, the overall content in disorder decreases in TtABA-WDS domain, as observed from the decrease in the amplitude of the negative peak at 200 nm. However, the structural transition is not comparable to that observed with glycerol, supporting that these two domains probably fold because hydrogen bonds with water molecules are replaced by intramolecular ones and not because of excluded volume effects.

### 2.8. TtABA-WDS and HvABA-WDS Confer Salt, Heat and Cold Stress Tolerance to Yeast Cells

The ASR gene family is absent in yeast cells [13]. In our previous work, we showed that overexpression of TtASR1 protein (cloned from Tunisian durum wheat landraces) increases *Saccharomyces cerevisiae* tolerance to salt and osmotic stress [9]. In a context where no functional characterization has yet been carried out for the ABA-WDS domain, the TtABA-WDS and HvABA-WDS were expressed in the yeast *Saccharomyces cerevisiae* to assess their possible protective effects. The cDNAs encoding the two domains were sub-cloned into the pYES2 expression vector downstream of the inducible GAL1 promoter. Yeast cells were transformed with pYES2 empty vector or with the two recombinant vectors bearing the insert encoding either TtABA-WDS or HvABA-WDS. The recombinant yeast cells were subjected to salt (2M NaCl), cold (4 °C) and heat (50 °C) stress.

**Figure 8:**
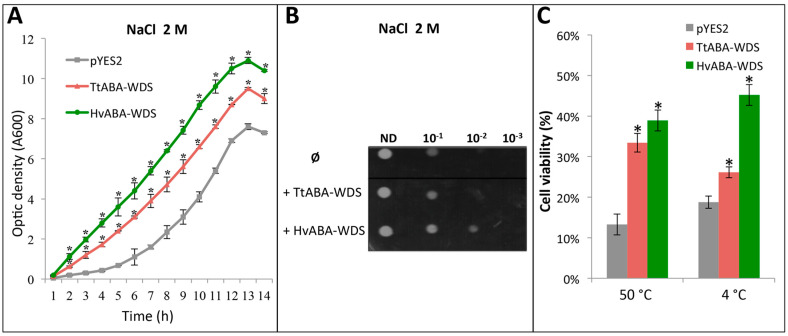
Expression of HvABA-WDS and TtABA-WDS confer salt, heat and cold tolerance to yeast cells. (**A**) Growth of induced yeast cultures transformed with the pYES2 plasmid (empty vector) or pYES2 derivatives encoding HvABA-WDS or TtABA-WDS in standard medium under salt stress (2M NaCl). The increasing density of the liquid cultures was measured at 600 nm. Values are the mean ± standard deviation (SD) from three independent samples. (**B**) Yeast strains transformed with pYES2 empty vector (Ø), pYES2 derivative encoding HvABA-WDS (+ HvABA-WDS) or TtABA-WDS (+ TtABA-WDS) were grown for 2 days under normal growth conditions (30 °C) or under salt stress (2M NaCl) in minimum solid media containing galactose as carbon source. Shown growth tests are representative of at least three independent replicates. ND: undiluted culture. 10^−1^, 10^−2^ and 10^−3^: 1:10, 1:100 and 1:1000 dilution of the culture. (**C**) Cell viability of yeast transformed with pYES2, pYES2-HvABA-WDS and pYES2-TtABA-WDS constructs under heat and cold stress. The values are the mean ± SD from three independent samples. The asterisks in the absorbance and cell viability indicate statistical significant differences (< 0.05) with respect to the control strain as evaluated with Student’s T test.

After transformation, growth assays of yeast cells on solid and liquid media in the presence of galactose and salt (2M NaCl) were conducted. Under salt stress the recombinant yeast strains expressing HvABA-WDS and TtABA-WDS domains grow better than the control strain bearing an empty vector, with HvABA-WDS conferring a higher resistance to salt stress compared to TtABA-WDS in both liquid and solid media (Figure 8A,B).

In addition, expression of the two domains in yeast increases cell viability under extreme temperatures (Figure 8C). The overexpression of the HvABA-WDS and TtABA-WDS domains leads to a 2.5-fold and 2.9-fold increase, respectively, in the number of CFUs under heat stress compared to the control strain bearing the empty vector. Likewise, the two domains exert a protective effect against cold stress. The overexpression of TtABA-WDS and HvABA-WDS confers a 1.3-fold and 2.4-fold increase, respectively, in cell viability compared to the control strain (Figure 8C).

In conclusion, yeast cells transformed with plasmids driving the expression of TtABA-WDS and HvABA-WDS are less sensitive to abiotic stress (salt, cold and heat) compared to cells transformed with an empty vector, indicating that the ABA-WDS domains are expressed and functional in yeast cells and confer them abiotic stress tolerance.

### 2.9. HvABA-WDS and TtABA-WDS Domains Stabilize the Enzyme LDH against Thermal and Dessication Stress

**Figure 9:**
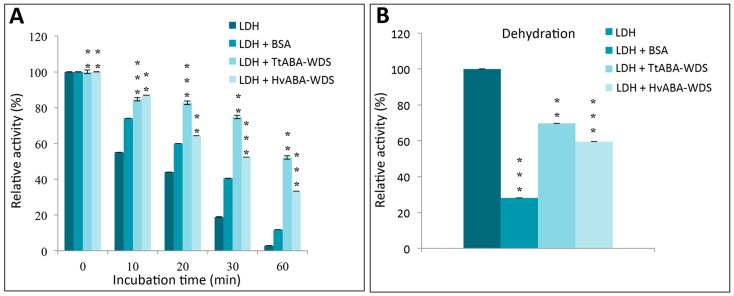
HvABA-WDS and TtABA-WDS domains protect LDH activity under heat stress and dehydration. (**A**) LDH was heated at 43 °C for the specified time in the absence or presence of HvABA-WDS and TtABA-WDS purified domains. A small aliquot was taken at each time point, and LDH activity was measured. (**B**) Residual LDH activity after dehydration and rehydration in the presence or absence of BSA, HvABA-WDS or TtABA-WDS. Data are presented as relative activity (%) with respect to the LDH activity recorded before stress treatment. The values are the mean ± SD from three independent samples. The asterisks denote statistically significant differences (** *p* < 0.01, *** *p* < 0.001) as evaluated with Student’s T test.

To ascertain whether the HvABA-WDS and TtABA-WDS domains have a chaperone activity in vitro, we assessed their ability to protect LDH activity under heat and dehydration stress, as already described for ASR proteins from tomato [48], banana [13], lily [49] and durum wheat [9]. The protective effects exerted by the two domains were compared to those observed either in the presence of BSA, as an example of a non-specific protectant, or in the absence of any protein. In the presence of BSA, LDH gradually loses its activity, with its residual activity being about 11% after 60 min at 43 °C (Figure 9A). By contrast, heat inactivation of LDH is significantly reduced in the presence of HvABA-WDS and TtABA-WDS with 33% and 52% of its initial activity being retained, respectively, after 60 min at 43 °C (Figure 9A). After dehydration and rehydration, the LDH activity was reduced to 28% of its initial value. TtABA-WDS and HvABA-WDS domains were found to confer a protective effect against dessication-induced enzyme inactivation, with the LDH residual activity reaching values of ~70% and 60%, respectively (Figure 9B). Results indicate that TtABA-WDS provides a higher degree of protection against thermal and dessication-induced denaturation compared to HvABA-WDS.

## 3. Discussion

Experimental information on the conserved ABA-WDS domain of the ABA-WDS family (Pfam 02496) in isolation is lacking. To fill this gap in knowledge, we herein isolated from stressed leaves the cDNAs encoding two ABA-WDS domains, one from durum wheat and one from barley. Using various bioinformatics approaches, we showed that these domains are predicted to be intrinsically disordered, and further confirmed their disordered nature experimentally. Analysis of their deduced amino acid sequence revealed a biased composition, i.e., an enrichment in disorder-promoting amino acids (A, H, and E) as reported for most ASR [17] and LEA proteins [21]. Many Pfam domains contain sequences with 100% predicted intrinsic disorder [50]. Noteworthy, the Pfam database recently added “disordered” as a specific entry type, underscoring the importance of disorder in the definition of protein domains [51]. Illustrative examples of proteins with IDDs playing key regulatory roles in controlling the cell cycle are p27kip1, p21Waf1/Cip1/Sdi, p57kip2 [52,53] and p53 [54]. Many of the signaling pathways and regulatory systems in eukaryotic cells are controlled by proteins bearing IDDs that mediate specific protein–protein and protein–nucleic acid interactions. As an example, TFs contain a high fraction of functionally essential intrinsically disordered regions (IDRs) [55]. In this context, direct and indirect evidence that ASR proteins can function as TFs [6,8] does not come as a surprise.

Although the two ABA-WDS domains are predicted to be disordered, they slightly differ in their predicted disorder content, with HvABA-WDS being predicted to be slightly more ordered than its cognate TtABA-WDS domain. This subtle predicted difference was experimentally confirmed by spectroscopic (i.e., CD) and hydrodynamic (i.e., SEC) studies. The ellipticity values at 200 and 222 nm, as observed in the far-UV CD spectra, enabled classifying TtABA-WDS and HvABA-WDS within the RC-like and PMG-like subfamilies, respectively. Slight differences between the two domains are also observed in their near-UV CD spectra. In line with spectroscopic data, the CI values, calculated from SEC, spotlight conformational differences between the two domains and showed that HvABA-WDS is more compact than TtABA-WDS. This slight difference in disorder content could result from subtle sequence differences (see Figure 1A). Although the two domains exhibit a high sequence identity (~94%), the presence of a valine in HvABA-WDS that replaces Ala17 in TtABA-WDS entails the formation of a bigger hydrophobic cluster in HvABA-WDS (Figure 2A) that may explain the comparatively higher order-propensity of the latter. It should be pointed out that conformational differences have the potential to also impact function, as well exemplified for instance by ubiquitin E3 ligases whose ubiquitination activity was shown to be reduced by amino acid substitutions inducing a more extended shape [56].

The differences in compactness and in the extent of secondary structure between the two domains mirror differences in their folding abilities. Indeed, while TtABA-WDS undergoes temperature-induced folding, as already observed for other IDPs [57,58], HvABA-WDS does not. The lack of effect of temperature on the secondary structure content of HvABA-WDS might arise from the fact that this domain is already more structured compared to TtABA-WDS. Interestingly, this differential behavior of the two domains vis-à-vis temperature mirrors to some extent the behavior we previously observed for the ASR proteins from durum wheat (TtASR1) and from barley (HvASR1), where the latter was found to undergo a temperature-induced folding less pronounced than that of the cognate ASR protein from durum wheat in spite of the high sequence similarity between the two proteins [17].

Many studies showed that various mechanisms trigger a gain of the structure by IDPs/IDDs, including disulfide bond formation, ion coordination, macromolecular partner binding and many others [59]. Taking into account the role of ABA-WDS domains in plant abiotic stress tolerance, it is conceivable that environmental changes may induce their folding. In line with this, in a previous work, we showed that TtASR1 and HvASR1 gain structure upon the addition of TFE, glycerol and zinc ions [17]. In the presence of TFE, a co-solvent commonly used in folding studies, both HvABA-WDS and TtABA-WDS domains gain α-helical structure. The two domains also undergo a disorder to α-helix transition upon glycerol-induced water deficit stress. Notably, while the CD spectra obtained in presence of TFE show an isodichroic point reflecting a two-state transition (Figure 6), a more complex scenario is observed in the presence of glycerol. In this case indeed, while the CD spectra of TtABA-WDS display an isodichroic point, in the case of HvABA-WDS only the CD spectra obtained in the 10–40% glycerol range do exhibit an isodichroic point (Figure 7) suggesting that distinct structural transitions could take place at 50% and that the glycerol-induced structural transition experienced by this domain is more complex than a simple two-state transition.

The subtle differences in terms of disorder content and folding propensity between the two domains parallel those previously observed with the ASR proteins from durum wheat (TtASR1) and barley (HvASR1) [17]. These ASR proteins, which encompass an ABA-WDS domain distinct, though similar to the domain herein reported (see Supplementary Figure S4), were indeed shown to feature slight conformational differences, with HvASR1 being more ordered than TtASR1 [17].

The fact that even as few amino acid differences as those observed between the two ABA-WDS domains engender measurable differences in their conformational properties and folding abilities, illustrates how the amino acid sequence finely tune protein behavior and properties. In addition, it should be emphasized that the conformational differences between the two domains could be hardly anticipated in light of the conservative nature of the amino acid substitutions that they bear, which further underscores the value of experimental approaches.

The abundance of histidine residues in the HvABA-WDS (12.1%) and TtABA-WDS (13.6%) domains provides a hint of their possible ability to bind bivalent cations such as zinc. We previously showed that the HvASR1 and TtASR1 proteins fold upon binding to zinc and mapped the region that undergoes Zn-induced structural transition [17]. This region contains a motif (PEHAHKHK) that was shown to be responsible for binding to zinc in tomato ASR1 [60]. This motif is also conserved in the ABA-WDS domains herein described (see Figure 1A and Supplementary Figure S4), thus further supporting their expected ability to bind zinc. The metal binding abilities of these domains and their impact on their conformational properties are the focus of ongoing studies that, ultimately, are expected to enable establishing correlations with their functions and possible transactivation capabilities.

Although the precise physiological function of the ASR gene family remains elusive, ASR proteins have been shown to have chaperone-like activity for direct plant protection under stress [3,7,9]. We recently reported that the ASR protein durum wheat (TtASR1) can function as a chaperone-like protein and increases *Sacchaomyces cerevisiae* tolerance under salt and osmotic stress [9]. In light of the fact that no functional characterization has yet been carried out for the ABA-WDS domain in isolation, we resorted to investigating the functional impact of TtABA-WDS and HvABA-WDS domains both in vitro and in vivo. *S. cerevisiae* is a widely used model for the functional characterization of stress-associated proteins. Here, we show that the heterologous expression of TtABA-WDS and HvABA-WDS domains increase *S. cerevisiae* viability under cold, heat and salt stress. The yeast growth assays carried out under stress conditions show that HvABA-WDS has a more pronounced protective effect compared to TtABA-WDS. Although these functional differences may be tied to the subtle differences observed in the structural and folding properties of these two domains, the possibility that the lower efficiency of TtABA-WDS in conferring tolerance to abiotic stress to yeast might arise from a lower expression level compared to HvABA-WDS cannot be ruled out. Definitive conclusions await future studies that are currently in progress in our lab.

LDH activity assays confirmed and extended the results obtained in vivo and showed that the TtABA-WDS and HvABA domains are able to protect the sensitive LDH enzyme from heat and dehydration in vitro. While HvABA-WDS was found to be more effective in protecting yeast against abiotic stress than TtABA-WDS, the opposite scenario is observed in vitro. The discrepancy between the in vitro and the in vivo results may arise from the expectedly higher complexity of chaperone activity in vivo, where the function of chaperones can potentially rely on various co-chaperones (e.g., proteins, membrane, DNA …). Moreover, ASR proteins were shown to function as transcriptional factors in addition to their chaperone activity. The in vivo abiotic stress tolerance phenotype is the result of all these combined activities.

The finding that the ABA-WDS domains have a chaperone activity is in line with the well-established relationship between intrinsic disorder and chaperone function [61]. Many chaperones are either fully disordered or contain IDRs that are involved in the regulation of the chaperone or in the interaction with the substrate itself [62]. Interactions mediated by IDRs of chaperones allow them to assist the folding of a much broader range of substrates. In other words, the lack of structure of chaperones is instrumental for their chaperone activity. In support of this, the chaperone activity of tomato ASR1 is not enhanced by the gain of structure induced by Zn^2+^ [19]. The conformational plasticity of the ABA-WDS domain could explain the multiple physiological functions attributed to the proteins of the ASR family. Additional experiments will be needed to unravel the relationships between the conformation adopted by the ABA-WDS domains under various stress conditions and their diverse biological functions. 

Although awaiting validation in planta, the in vivo assays in yeast and the in vitro LDH protective assay provide evidence for the implication of the ABA-WDS domain in the known function attributed to the ASR family and advocate for the potential use of the ABA-WDS domain in biotechnological applications.

Finally, the present study contributes to shedding light on the hitherto ambiguous classification of ASR proteins. Their biased amino acid composition constitutes one of the pillars onto which relies on the previously proposed classification of the ASR protein family within the LEA superfamily [23,24]. In light of the lack of sequence similarity with any of the recognized LEA group proteins, Hunault and Jaspard proposed however the exclusion of ASR proteins from the LEA family [26]. While waiting for more extensive studies, the results herein presented would advocate in favor of a reconsideration of the ABA-WDS family as a member of the LEA superfamily. In fact, the structural and functional characterization of the wheat and barley domains unveiled that beyond a biased composition, they share most of the known features of LEA proteins, i.e., (i) they are intrinsically disordered, (ii) they undergo α-helical folding under desiccation, and (iii) they are endowed with a heat, desiccation and cold protective activity and their overexpression in yeast confer abiotic stress tolerance.

## 4. Materials and Methods

### 4.1. Plant Materials and Stress Treatments

The local Tunisian genotype of tetraploid wheat *T. turgidum* L. subsp. durum (2n = 4x = 28) cv. Mahmoudi and *Hordeum vulgare* L. (Rihane), were used for ABA_WDS cDNA cloning. All seeds were initially surface sterilized by a 0.5% NaOCl wash for 15 min, rinsed three times with sterile water, and germinated on wet Whatman paper filter placed in Petri dishes after 2 days in the dark. Ten-day-old seedlings grown were subjected to stress. For salinity seedlings were incubated in 200 mM NaCl. Stressed seedlings were sampled at 24 h of treatment. Leaves were harvested and frozen in liquid nitrogen for RNA isolation.

### 4.2. RNA Extraction and Amplification of TtABA-WDS and HvABA-WDS cDNA Domains from Durum Wheat and Barely

RNA was isolated from approximately 200 mg of durum wheat and barley leaves according to the Trizol method (Invitrogen) and following the manufacturer’s instructions. RNA was treated with RNase-free DNase to remove any contaminating DNA. Reverse transcription reactions were performed for 1 h at 37 °C using MML-reverse transcriptase (Invitrogen) and oligo-dT. First-strand complementary DNA (cDNA) was used as a template for PCR amplifications using a couple of specific primers (PD1-PD2) designed from the alignment of a set of full ASR protein sequences belonging to monocot plant species [9]. Control amplifications in the absence of the reverse transcriptase were also performed to rule out any amplification caused by the presence of contaminating DNA.

### 4.3. Cloning, Expression, and Production of Recombinant TtABA-WDS and HvABA-WDS cDNA in E. coli

The TtABA-WDS and HvABA-WDS cDNA were cloned into the EcoRI site of the *E. coli* expression vector pGEX-4T-1, resulting in a fusion with glutathione S-transferase (GST). The two domains were expressed and purified as previously described [17]. Removal of the GST tag by thrombin results in a recombinant product bearing a vector-encoded N-terminal GSPEF and a C-terminal EFPGRLERPHRD amino acid extension. Disorder content analysis of the resulting recombinant products (Supplementary Figure S1C,D) indicates that the presence of the vector-encoded residues preserves (actually slightly increases) the predicted disordered nature of the domains, in line with the expectedly disordered nature of vector-encoded linkers.

Protein concentrations were calculated using the theoretical absorption coefficients at 280 nm as obtained using the program ProtParam at the EXPASY server (https://web.expasy.org/protparam/ (accessed on 25 January 2021)).

### 4.4. Mass Spectrometry

#### 4.4.1. Intact Protein Mass Analysis

Protein masses were determined on purified solution samples. An amount of 1 μL of protein at ~20 μM was mixed with 1 μL of α-Cyano-4-hydroxycinnamic acid matrix (Bruker Daltonics, Wissembourg, France) solution in 0.3% TFA/CH3CN (50:50 *v*/*v*). One μL of the mix was spotted on the target and analysed by MALDI-TOF on an Ultraflex III spectrometer (Bruker Daltonics, Wissembourg, France) controlled by the Flexcontrol 3.0 package (Build 51) and operated in the linear mode, using a maximum accelerating potential of 25 kV and a 5000–15,000 *m*/*z* range (LP_Protmix_Method). The laser frequency was fixed to 100 Hz and ~1000 shots per sample were cumulated. Four external standards (Protein Calibration Standard I, Bruker Daltonics, Wissembourg, France) were used to calibrate each spectrum to a mass accuracy within 100 ppm. Peak picking was performed using the FlexAnalysis 3.0 software with an adapted analysis method. Parameters used were: centroid peak detection algorithm, S/N threshold fixed to 5 and a quality factor threshold of 30.

#### 4.4.2. Peptide Mass Fingerprinting

Proteins in SDS-PAGE gel-excised bands were submitted to Glu-C digestion in 50 µL NH_4_HCO_3_ 25 mM supplemented with 12.5 ng/μL enzyme (V1651, Promega, Charbonnières-les-Bains, France) (37 °C, overnight). This sequencing grade serine protease (*S. aureus* V8) specifically cleaves at the C-terminus of either aspartic or glutamic acid residues. Peptide extracts collected from the digestion solution, from the first extract in formic acid 5%, and from the second one in formic acid 5%/CH_3_CN (40:60 *v*/*v*) were pooled and dried in a centrifugal vacuum system. Samples were reconstituted in TFA 0.1%/CH_3_CN (80:4 *v*/*v*) and analyzed by LC-MS/MS using an LTQ Orbitrap QExactivePlus mass spectrometer (Thermo Electron, Bremen, Germany) online with a nanoLC Ultimate 3000 chromatography system (Dionex, Sunnyvale, CA, USA). Peptides were first concentrated on a pre-column (C18 PepMap100, 2 cm × 100 μm, 100 Å, 5 μm, Dionex, Sunnyvale, CA, USA) in loading buffer (0.05% TFA/CH_3_CN (98:2 *v*/*v*)), then they were separated on a reverse-phase LC EASY-Spray C18 column (Acclaim PepMap RSLC C18, 15 cm × 75 μm, 100 Å, 2 μm, Dionex, Sunnyvale, CA, USA; flow rate 300 nL/min) equilibrated in 4% of solvent B (0.1% formic acid/CH_3_CN (20:80 *v*/*v*)) in solvent A (0.1% formic acid) and eluted using a linear gradient of 4–55% B in A in 30 min. All chemicals (TFA, CH_3_CN) are Mass Spectrometry Grade (Sigma-Aldrich, St-Quentin-Fallavier, France).

For peptide ionization in the EASY-Spray source, the spray voltage was set at 1.9 kV and the capillary temperature at 275 °C. The spectrometer was operated in the data-dependent mode to switch consistently between MS and MS/MS. MS spectra were acquired in the 300–1700 *m*/*z* range at full width at half maximum (FWHM) resolution of 30,000 measured at 400 *m*/*z*. For internal mass calibration, the 445.12 *m*/*z* ion was used as lock mass. The 10 most abundant precursor ions were selected and collision-induced dissociation fragmentation was performed in the ion trap to insure both maximum sensitivity and a maximum amount of MS/MS data. The signal threshold for an MS/MS event was set to 500 counts. Charge state screening was enabled to exclude precursors with 0 and 1 charge states. Dynamic exclusion was enabled with a repeat count of 1, exclusion list size of 500 MS runs and exclusion duration of 30 s. The acquired raw data were processed using Proteome Discoverer (version 1.4.1.14, Thermo Fisher Scientific). Spectra were searched using SEQUEST (Thermo Fisher Scientific) against a homemade database comprising 20,150 human and 4306 *E. coli* sequences and the TtABA-WDS and the HvABA-WDS sequences. To detect contaminants, an additional search against the Swiss-Prot database (https://www.uniprot.org/uniprot (accessed on 25 January 2021); version 2014.02; 20,284 entries) supplemented with a set of 245 frequently observed contaminants (e.g., human keratin) was performed using MASCOT (http://www.matrixscience.com (accessed on 25 January 2021)). Search parameters were: (i) GluC; five miscleavage allowed; (ii) mass tolerance of 6 ppm for monoisotopic precursor ions and 0.8 ppm for fragment ions from MS/MS; (iii) Cys carbamidomethylation (+57.02146 Da) as a fixed modification; Met oxidation (+15.99491 Da) and N-terminal acetylation (+42.0106 Da) as variable modifications; (iv) minimum peptide length of four residues. Only high-score peptides were selected. Proteins were identified with a false discovery rate (FDR) of 1%.

### 4.5. Size Exclusion Chromatography and Calculation of Hydrodynamic Radii

The hydrodynamic radii (Stokes radii, R_S_) of the TtABA-WDS and HvABA-WDS domains were estimated by analytical SEC as previously described [17]. Typically 2 mg mL-1 of purified protein was injected. The SEC buffer was PBS pH 7.3.

The Stokes radii of domains eluted from the SEC column were deduced from a calibration curve obtained using globular proteins of known molecular mass (MM, in Daltons) and whose RS (in Å) was calculated according to [63]:

log (R_S_^Obs^) = 0.369 × (log MM) − 0.254 (1)

The R_S_ (in Å) of a natively folded protein (R_s_N^F^) and of a fully unfolded state in urea (R_S_U) with a molecular mass (MM) (in Daltons) were calculated according to [64]:

log (R_S_^NF^) = 0.357 × (log MM) − 0.204 (2)

log (R_S_^U^) = 0.521 × log (MM) − 0.649 (3)

The R_S_ of an IDP with N residues was also calculated according to [65] using the simple power-law model:

R_S_^IDP^ = R_0_N^ν^ (4)

where R_0_ = 2.49 and ν = 0.509.

The compaction index (CI) is expressed as according to [66]:

CI = (R_s_^U^ − R_s_^obs^)/(R_s_^U^ − R_s_^NF^)) (5)

This parameter, which allows comparison between proteins of different lengths, increases with increasing compaction and, in principle, varies between 0 and 1.

### 4.6. Differential Scanning Fluorimetry (DSF)

The DSF analysis of TtABA-WDS and HvABA-WDS domains was carried out in the presence of a fluorescent dye as already described [17]. This experiment was conducted using a PCR instrument (Biorad) and 96-well plates containing 25 µL of mixture per well. Each well contained 21.5 µL of TtABA-WDS domain or HvABA-WDS domain at 1 mg mL^−1^ in solution and 3.5 µL of SYPRO Orange solution (from a 5000× stock solution). Fluorescent signals were acquired with excitation and emission wavelengths at 485 nm and 625 nm, respectively. Temperature scans were performed from 20 °C to 90 °C.

### 4.7. Circular Dichroism (CD) Measurements

The CD spectra of TtABA-WDS and HvABA-WDS were recorded using a Jasco 810 dichrograph, flushed with N2 and equipped with a Peltier thermoregulation system [17]. One-mm or 1-cm thick quartz cuvettes were used for far- and near-UV CD measurements, respectively. Proteins concentrations were 0.12 mg mL^−1^ and 1.2 mg mL^−1^ for far- and near-UV CD studies, respectively. Far-UV CD spectra were measured between 190 and 260 nm, while near-UV CD spectra were recorded between 250 and 350 nm. Unless differently specified, CD spectra were recorded in 10 mM sodium phosphate pH 7 at 20 °C. The scanning speed was 20 nm/min, with data pitch of 0.2 nm. Each spectrum is the average of three acquisitions. The spectrum of buffer was subtracted from the protein spectrum. Spectra were smoothed using the “means-movement” smoothing procedure implemented in the Spectra Manager package.

Far-UV CD spectra were also recorded in the presence of increasing concentrations of TFE (from 10% to 50%), glycerol (from 10% to 50%) and sucrose (0% and 30%).

Mean molar ellipticity values per residue (MRE) were calculated as (Θ) = 3300 MΔA/(lcn), where l is the pathlength in cm, n is the number of residues, M is the molecular mass in Daltons and c is the concentration of the protein in mg mL^−1^. Numbers of amino acid residues are 83 for both domains. The molecular mass is 9200 Da and 9234 Da for TtABA-WDS and HvABA-WDS, respectively.

Temperature-induced protein folding events were assessed by measuring the ellipticity at a fixed wavelength of 230 nm in the temperature range of 20–80 °C, with data pitch 2 °C and a temperature slope of 2 °C/min and protein concentrations of 0.12 mg mL^−1^. The reversibility of the process was assessed by monitoring the ellipticity during cooling from 80 °C to 20 °C.

The DICHROWEB website (http://dichroweb.cryst.bbk.ac.uk/html/home.shtml (accessed on 25 January 2021)), which was supported by grants to the BBSRC Centre for Protein and Membrane Structure and Dynamics (CPMSD) [67], was used to analyze the experimental data in the 190–260 nm range. The content in the various types of secondary structure was estimated using the CONTIN deconvolution method with the reference protein set 7 [68].

For the estimation of the percentage of residues adopting an α-helical conformation in the presence of various additives (TFE, glycerol, and sucrose) we analyzed the value of molar ellipticity at 220 nm observed under each condition and divided it by the value expected for a protein in which all residues adopt an α-helical conformation (i.e., 100% α-helix). The latter was calculated according to the following empirical relationship [69]:

Θ _220_ = −39,500 × [1 − (2.57/n)] (6)

where n is the number of residues.

### 4.8. Salt Tolerance and Growth Assays of Yeast Cells

The effect of salt stress on the growth of yeast strain W303-1B (MATα; ade2-1; ura3-1; his3-11,15; leu2-3112; trp1-1; can1-100) of *S. cerevisiae* harboring recombinants plasmids (TtABA-WDS or HvABA-WDS) or empty vector (pYES2) was studied. The pYES2 expression vector (Invitrogen) is a 2 μ based multicopy plasmid and contains the URA3 gene and the Gal1 promoter for selection and expression in yeast. Stationary yeast precultures grown on glucose-containing minimal medium (MM) were diluted to 5 × 106 cells mL^−1^ and used to inoculate (1:100) yeast cultures on MM Gal in the absence or presence of NaCl (2M) under continuous agitation (200 r.p.m.) at 30 °C. The OD at 600 nm was measured every 2 h. For kinetic studies on solid medium, serial dilutions of different recombinant yeast clones were prepared (10^−1^–10^−3^) from the same precultures, and then 7 µL from each dilution were spotted onto solid MMGal containing NaCl (2M) and incubated at 30 °C for three days. Cell viability was estimated by counting the number of colony-forming units after incubation of the plate overnight at 30 °C. The viability ratio under salt stress was calculated according to the following formula: (mean of colony number on a stressed plate/mean of colony number on a control plate) × 100%.

### 4.9. LDH Protective Assay

The activity of lactate dehydrogenase (LDH, EC1.1.1.27, rabbit muscle lactate dehydrogenase) was measured as previously described [9]. One µL of a solution of LDH at 20 μg/mL (Sigma, Tokyo, Japan) was added to a 20 µL-solution containing 20 μg/mL of either bovine serum albumin (BSA), or TtABA-WDS or HvABA-WDS in 10 mM sodium phosphate, pH 7.4. Samples were then submitted to heat stress treatment. To test the effect of desiccation on LDH activity with or without TtABA-WDS or HvABA-WDS, samples were vacuum-dried in a Speed Vac (Concentrator plus / Vacufuge^®^ plus of Eppendorf AG, Hamburg, Germany) to a final volume of 6 µL and rehydrated by the addition of 14 µL of water.

To determine the LDH activity, 20 μL of the LDH mixture was added to 980 μL of assay buffer (10 mM sodium phosphate, pH 7.4, 2 mM NADH, and 10 mM pyruvic acid). NADH oxidation was monitored by measuring the absorbance at 340 nm over 3 min, during which the reaction rate was linear. The rate of absorbance was then used to calculate the activity ΔA340/min × 8095 = U/L (Biomaghreb kit, Biomaghreb, Tunis, Tunisia). All samples were assayed in triplicate.

### 4.10. Bioinformatics Analyses

Identification of the conserved domain of TtABA-WDS and HvABA-WDS was carried out by query against the Conserved Domain Database (CDD) (http://www.ncbi.nlm.nih.gov/Structure/cdd/wrpsb.cgi (accessed on 25 January 2021)). The ExPASy server (http://web.expasy.org/protparam/ (accessed on 25 January 2021)) was used to derive the physicochemical properties of the domains. Sequence alignment of the TtABA-WDS and HvABA-WDS domains was performed using Clustal Omega (https://www.ebi.ac.uk/Tools/msa/clustalo/ (accessed on 25 January 2021)). The RH-plot was generated by the PONDR server (http://www.pondr.com (accessed on 25 January 2021)). The phase diagram plots of TtABA-WDS and HvABA-WDS were generated using the CIDER server (http://pappulab.wustl.edu/CIDER/analysis/ (accessed on 25 January 2021)). Deviations in amino acid composition of TtABA-WDS and HvABA-WDS were computed as previously described [17] using the average amino acid frequencies of the SWISS-PROT database (as obtained from https://web.expasy.org/protscale/pscale/A.A.Swiss-Prot.html (accessed on 25 January 2021)) as the reference value. The average amino acid frequencies of the SWISS-PROT database roughly correspond to the mean composition of proteins in nature. If the average composition of an amino acid X in SWISS-PROT proteins is CSPX, and CPX is the composition of X within a protein P, deviation from the composition of X of SWISS-PROT proteins was defined for P as (CPX-CSPX)/CSPX. The HCA plots and the IUPRED disorder prediction were obtained using the MeDor server [34]. Structure prediction was carried out using the Phyre2 protein fold recognition server (http://www.sbg.bio.ic.ac.uk/~phyre2/html/page.cgi?id=index (accessed on 25 January 2021)) which uses advanced remote homology detection methods to build 3D models [37].

## 5. Conclusions

In this work, two ABA-WDS domains of the ASR family were isolated from durum wheat and barley. These two domains were shown to be intrinsically disordered and to be able to undergo induced folding in the presence of TFE and glycerol, which mimic interaction and dehydration respectively. Strikingly, both domains confer stress resistance to yeast cells, as well as heat and dessication resistance to LDH in vitro. To the best of our knowledge, this is the first time a molecular function as a chaperone can be experimentally attributed to an ABA-WDS domain. Our results reveal that the ABA-WDS domain is a *bona fide* domain, capable of exerting its function in isolation, and whose conformational plasticity could underlie the functional versatility of the ASR protein family. The present results provide a foundation for further evolutionary and functional characterization of the ASR gene family, and contribute to enlarge our knowledge of this protein family. Beyond a fundamental interest, the present study also holds promise in the field of plant improvement. After an in planta validation step (which is currently in progress in our lab), the sequence encoding the ABA-WDS could be transferred to various crops to improve their tolerance to abiotic stress.

Lastly, and from a more general perspective, the data herein presented provide evidence that the ABA-WDS domain could be used to preserve enzyme activities under heat and cold stress thus paving the way towards possible biotechnological applications in the field of enzymology.

## Figures and Tables

**Table 1:** Properties of the deduced amino acid sequences of the TtABA-WDS and HvABA-WDS domains.

Proteins	N	MM	pI	f_+_	f_−_	FCR	NCPR	κ	GRAVY	PDR
TtABA-WDS	66	7192	6.12	0.136	0.197	0.333	−0.061	0.096	−0.904	2
HvABA-WDS	66	7226	6.24	0.151	0.197	0.348	−0.045	0.087	−0.851	2

N: residue number; MM: molecular mass in Daltons; f_+_: fraction of positively charged residues; f_−_: fraction of negatively charged residues; FCR: fraction of charged residues (f_+_ + f_−_); NCPR: net charge per residue, value of the difference between the fraction of positively charged and negatively positively residues, (|f_+_ − f_−_|); κ: linear distribution of opposite charges; GRAVY: grand average of hydropathy index (sum of hydropathy values of all amino acids divided by the protein length); PDR: phase diagram region.

**Table 2:** Stokes radii (R_S_^obs^, Å) as obtained by SEC and expected values for the various conformational states.

Protein	Mass (Da)	R_S_^obs^	R_S_^NF^	R_S_^U^	R_S_^IDP^	R_S_^obs^/R_S_^U^	R_S_^obs^/R_S_^IDP^	CI
TtABA-WDS	9200	21.5	16.2	26.1	23.6	0.83	0.91	0.46
HvABA-WDS	9234	20.3	16.2	26.1	23.6	0.78	0.86	0.59

R_S_^NF^: R_S_ expected for a natively folded (NF) form; R_S_^U^: R_S_ expected for a fully unfolded form; R_S_^IDP^: R_S_ expected for an IDP based on the simple power-law model; Mass: molecular mass calculated from the amino acid sequence of the recombinant protein. CI: compaction index.

## Data Availability

The TtABA-WDS and HvABA-WDS cDNA sequences were deposited in GenBank under accession numbers MT340098 and MT340099, respectively.

## Supplementary Materials

The following are available online at https://www.mdpi.com/1422-0067/22/5/2314/s1, Supplementary text on HCA. Figure S1: Charge-hydropathy plots, Figure S2: Results of Peptide Mass Fingerprint (PMF) of TtABA-WDS and HvABA-WDS domains, Figure S3: Far-UV CD spectra of TtABA-WDS and HvABA-WDS domains in the absence or presence of 30% sucrose, Figure S4: Amino acid sequence alignment of TtABA-WDS and HvABA-WDS domains with the ASR1 proteins from durum wheat (Tt) and barley (Hv).

## Abbreviations

ABA	Abscissic acid
ASR	Abscissic acid stress ripening protein
ABA_WDS	Abscissic acid water deficit stress domain
IDPs	Intrinsically disordered proteins
TF	Transcription factor
LEA	Late embryogenesis abundant protein
IDRs	Intrinsically disordered regions
IDDs	Intrinsically disordered domains
TFE	Trifluoroethanol
LDH	Lactate dehydrogenase
NaCl	Sodium Chloride
GST	Glutathione S-transferase
R_S_	Stokes radii
SEC	Size Exclusion Chromatography
DSF	Differential scanning fluorimetry
CD	Circular dichroism
BSA	Bovine serum albumin
CDD	Conserved Domain Database
