# Supplementary material for: Structural and Functional Characterization of the ABA-Water Deficit Stress Domain from Wheat and Barley: An Intrinsically Disordered Domain behind the Versatile Functions of the Plant Abscissic Acid, Stress and Ripening Protein Family
Source: Int J Mol Sci. 2021 Feb 26;22(5):2314. doi: 10.3390/ijms22052314 (PMC7956565; doi:10.3390/ijms22052314)
Supplement: Supplementary file 1 [file ijms-22-02314-s001.pdf]

Supplementary Information

Supplementary text

The hydrophobic cluster analysis (HCA) provides a graphical representation of the sequence that enables identifying disordered regions (Callebaut *et al.*, *Cell Mol Life Sci* 1997). Although HCA was not originally intended to predict disorder, it is very useful for unveiling disordered regions. HCA outputs can be obtained from http://mobyle.rpbs.univ-paris-diderot.fr/cgi-bin/portal.py?form=HCA#forms::HCA. HCA provides a two-dimensional helical representation of protein sequences in which hydrophobic clusters are plotted along the sequence. Glycine residues are represented as diamonds, prolines as stars, threonines as squares and serines as squares containing a dot. Acidic and basic residues are shown in red and in blue, respectively. Hydrophobic residues are shown in green. Disordered regions are recognizable as they are depleted (or devoid) in hydrophobic clusters. HCA also enables identifying short regions with propensity to fold that appear as regions locally enriched in small hydrophobic clusters within regions otherwise devoid of such clusters.

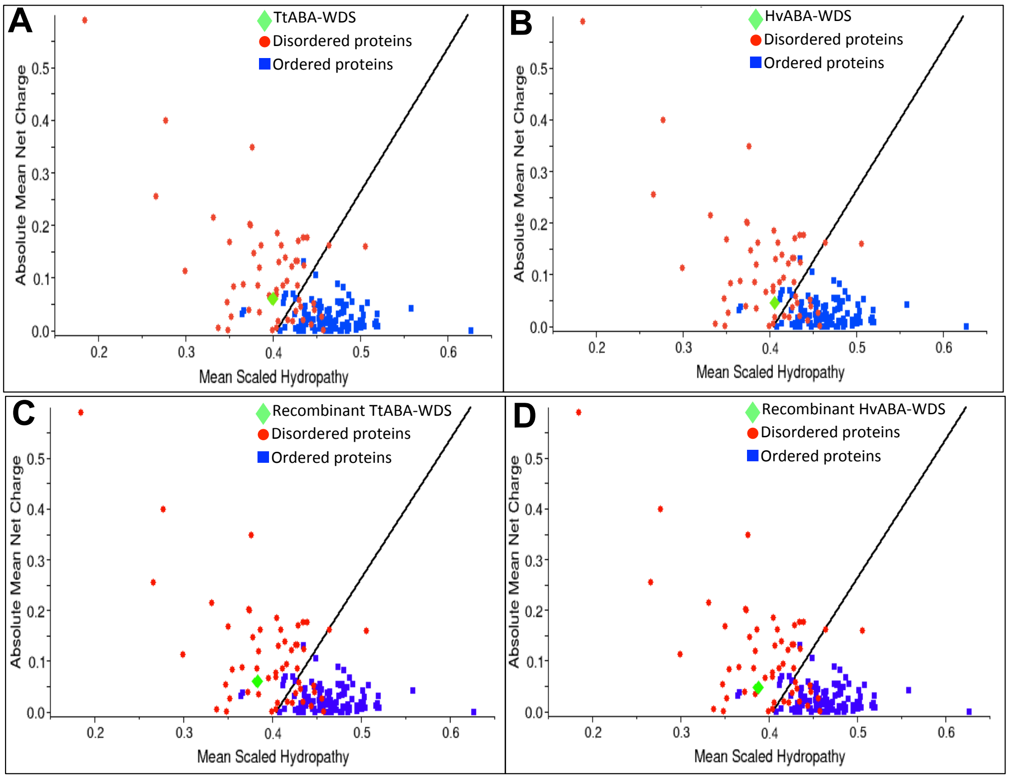

Figure S1. Charge-hydropathy plot of the native TtABA-WDS (**A**) and HvABA-WDS (**B**) domains and of the recombinant TtABA-WDS (**C**) and HvABA-WDS (**D**) domains including the non-native, vector-encoded amino acid residues. The mean net charge (R) is plotted against the mean hydrophobicity (H). In the left part of the CH plot, a protein is predicted to be intrinsically disordered, whereas it is predicted to be structured if it falls in the right part of it (see Materials and Methods).

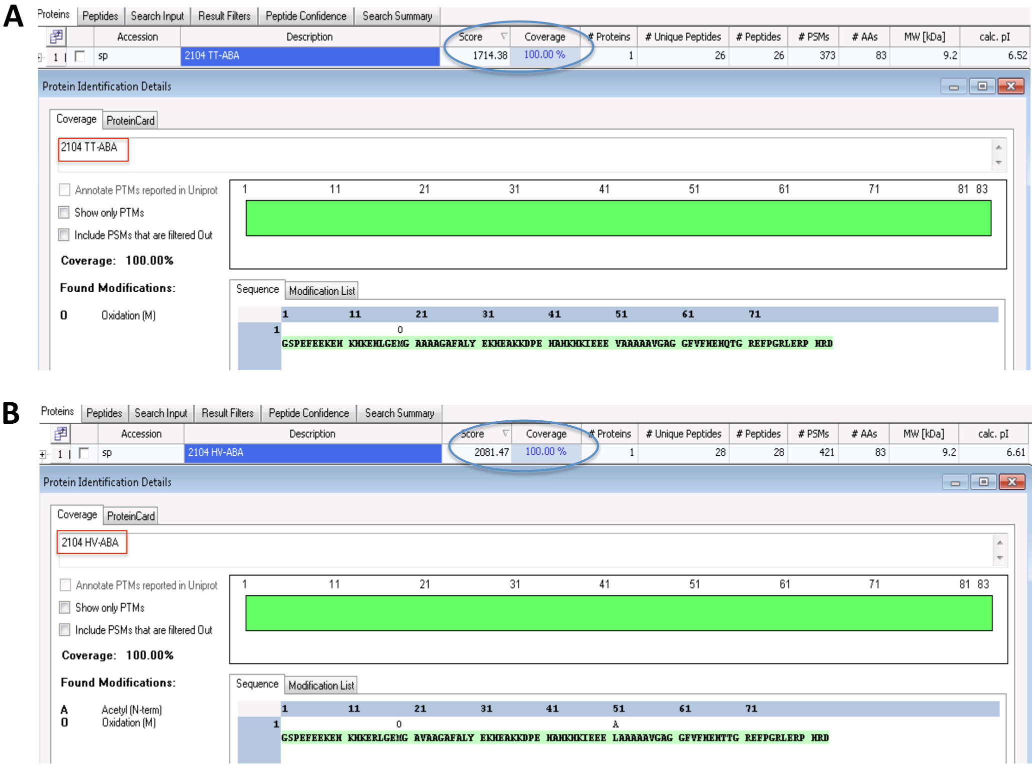

Figure S2. Results of Peptide Mass Fingerprint (PMF) of TtABA-WDS (**A**) and HvABA-WDS (**B**) domains. Peptides obtained by GluC enzymatic digestion are shown in green. The sequence coverage is 100 % for both domains (blue circle).

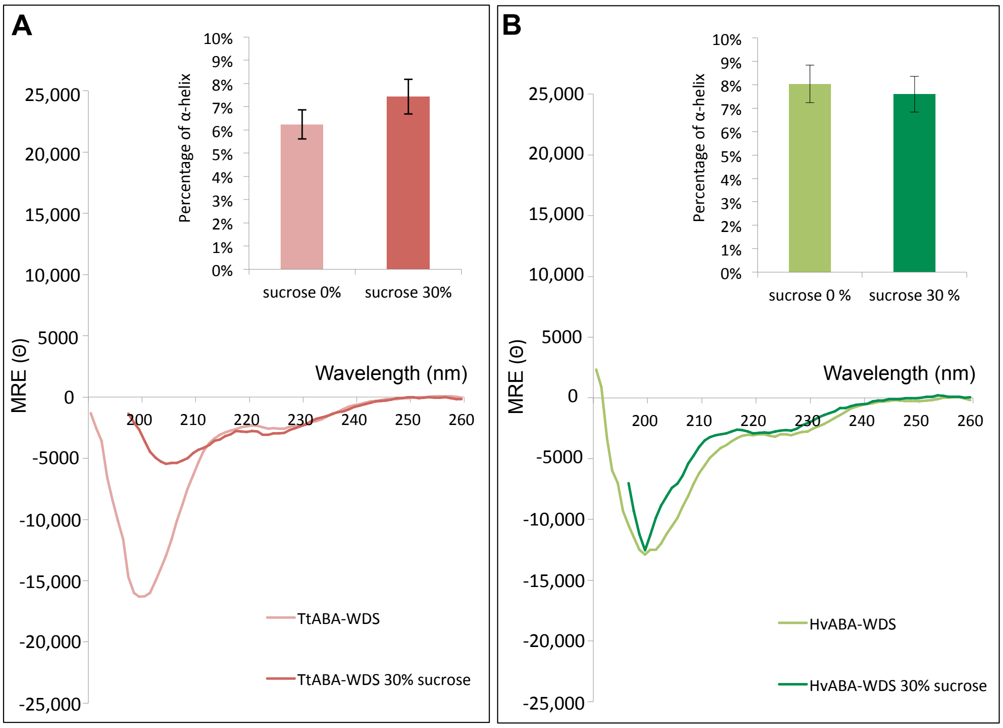

Figure S3. Far-UV CD spectra of TtABA-WDS (**A**) and HvABA-WDS (**B**) domains in the absence or presence of 30% sucrose at 20°C. Domains were at 0.12 mg/mL in 10 mM sodium phosphate at pH 7.

```
                1         10        20        30        40        50        60        70
                .         .         .         .         .         .         .         .
TtASR1      MAEEKHHHLFHHKEGEDFQPAADGGVDTYGYSTETVVTATGNDGEYERITKEEKHHKHKEHLGEMGAAAA
TtABA-WDS   ---------------------------------------------------EEKEHKHKEHLGEMGAAAA
                                                               ***:***************

                71        80        90        100       110       120       130
                .         .         .         .         .         .         .
TtASR1      GAFALYEKHEAKKDPEHAHKHKIEEEVAAAAAVGAGGFVFHEHHEKKQDHKEAKEASGEKKHHLFG
TtABA-WDS   GAFALYEKHEAKKDPEHAHKHKIEEEVAAAAAVGAGGFVFHEHQTGR
            *******************************************::::


                1         10        20        30        40        50        60        70
                .         .         .         .         .         .         .         .
HvASR1      MAEEKHHHHLFHHKKEGEDFQPAADGGADVYGYSTETVVTGTGNEGEYERITKEEKHHKHKEHLGEMGAV
HvABA-WDS   -----------------------------------------------------EEKEHKHKERLGEMGAV
                                                                 ***:*****:*******
71        80        90        100       110       120       130
                .         .         .         .         .         .         .
HvASR1      AAGAFALYEKHEAKKDPEHAHKHKIEEELAAAAAVGAGGFVFHEHHEKKQDHKEAKEASGEKKHHLFG
HvABA-WDS   AAGAFALYEKHEAKKDPEHAHKHKIEEELAAAAAVGAGGFVFHEHTTGR
            *********************************************::::
```

Figure S4. Amino acid sequence alignment of TtABA-WDS and HvABA-WDS domains with the ASR1 proteins from durum wheat (Tt) and barley (Hv) that we previously reported (Hamdi *et al.*, *Sci Rep* 2017). The amino acid sequences of the ABA-WDS domains are shown in red, while the sequences of the ASR1 proteins are shown in black. Amino acid substitutions are shown in bold on a yellow background. Asterisks denote identical amino acids, while colons indicate non-conserved residues. The alignment was generated with Clustal Omega and then edited manually.
